# β-Glucan Production by *Levilactobacillus brevis* and *Pediococcus claussenii* for In Situ Enriched Rye and Wheat Sourdough Breads

**DOI:** 10.3390/foods10030547

**Published:** 2021-03-06

**Authors:** Julia A. Bockwoldt, Johanna Fellermeier, Emma Steffens, Rudi F. Vogel, Matthias A. Ehrmann

**Affiliations:** Lehrstuhl für Technische Mikrobiologie, Technische Universität München, 85354 Freising, Germany; julia.bockwoldt@tum.de (J.A.B.); johanna.fellermeier@tum.de (J.F.); emma.steffens@tum.de (E.S.); rudi.vogel@tum.de (R.F.V.)

**Keywords:** EPS, β-glucan, sourdough, LAB, temperature effects, high performance liquid chromatography (HPLC), ELISA, sourdough bread, sensory evaluation

## Abstract

Sourdough fermentation is a common practice spread across the globe due to quality and shelf life improvement of baked goods. Above the widely studied exopolysaccharide (EPS) formation, which is exploited for structural improvements of foods including baked goods, β-glucan formation, by using lactic acid bacteria (LAB), offers additional values. Through renunciation of sucrose addition for bacterial β-d-glucan formation, which is required for the production of other homopolysaccharides, residual sweetness of baked goods can be avoided, and predicted prebiotic properties can be exploited. As promising starter cultures *Levilactobacillus* (*L.*) *brevis* TMW (Technische Mikrobiologie Weihenstephan) 1.2112 and *Pediococcus* (*P.*) *claussenii* TMW 2.340 produce *O*2-substituted (1,3)-β-d-glucan upon fermenting wheat and rye doughs. In this study, we have evaluated methods for bacterial β-glucan quantification, identified parameters influencing the β-glucan yield in fermented sourdoughs, and evaluated the sourdough breads by an untrained sensory panel. An immunological method for the specific detection of β-glucan proved to be suitable for its quantification, and changes in the fermentation temperature were related to higher β-glucan yields in sourdoughs. The sensory analysis resulted in an overall acceptance of the wheat and rye sourdough breads fermented by *L.* *brevis* and *P.* *claussenii* with a preference of the *L. brevis* fermented wheat sourdough bread and tart-flavored rye sourdough bread.

## 1. Introduction

Food fermentation by bacteria and yeasts is longer practiced than we know about the existence of microbes. Various lactic acid bacteria (LAB), including lactobacilli, lactococci, and pediococci, are exploited and deliberately applied as starter cultures in a broad range of safe fermented food, e.g., in the dairy and meat industry, for vegetables, baked goods, and alcoholic beverages [1,2]. Sourdough fermentation as one of the oldest biotechnological processes offers beneficial effects for the bread producers and consumers as the products have improved sensory qualities and shelf life [3,4,5]. The formation of exopolysaccharides (EPS) by LAB during sourdough fermentation offers additional values due to improved water binding capacity and the associated prebiotic potential [6,7,8,9,10,11,12]. Moreover, EPSs are discussed to positively influence human’s health, e.g., as prebiotics acting as fermentable substrates for the intestinal microbiota, immunoregulatory effects, reducing serum cholesterol levels, and lowering postprandial blood glucose and insulin response [13,14,15,16,17].

EPSs produced by LAB are either homopolysaccharides (HoPS) such as β-d-glucans, α-d-glucans, and β-fructans, formed by the same monosaccharide units or heteropolysaccharides (HePS), which are mainly composed of D-glucose, D-galactose, and L-rhamnose. Most HoPS are polymerized by extracellular glucansucrases (alteran, dextran, reuteran, or mutan) or fructansucrases (levan and inulin) from sucrose as the substrate [9,14,18,19,20]. In contrast, β-d-glucans and HePS formation proceeds by intracellularly synthesis of nucleotide-activated sugar moieties and subsequent polymerization by glycosyltransferases (*gtf*) [21,22,23,24,25]. By comparing both EPS types produced by LAB, extracellularly produced EPSs reach much higher yields (≤16 g/kg dough) than intracellular produced EPSs with yields below 0.6 g/L medium under optimal culture conditions [6,26]. It is previously reported that EPS formed by glucansucrase activity from sucrose during sourdough fermentation beneficially affects the viscoelastic properties, the texture, and shelf life of the dough [6].

In this case, however, sucrose addition is mandatory for HoPS synthesis and may lead to surplus acetate in the sourdough upon formation of glucan and utilization of the remaining fructose as an electron acceptor. In addition, residual sucrose from such sweet sourdoughs frequently results in sweet baked goods. In contrast, for β-d-glucan formation, the available soluble carbohydrates in wheat (≤1.85% maltose, sucrose, glucose, and fructose) and rye (≤2.0% maltose) flour are sufficient and, therefore, meet the demands of clean-label products [27,28,29,30,31,32,33,34]. Even low quantities of β-glucan have effects due to a network formed by EPS-encapsulated cells increasing the viscosity of liquid media, wheat sourdough, oat ferment, or spoiled beer [12,22,30]. Besides techno-functional properties of β-glucan from LAB, its health beneficial effects are gaining more attention, e.g., regulations of the blood cholesterol level, anti-inflammatory effects, and the stimulation of probiotic microorganisms [11,35,36].

The brewery isolates *Levilactobacillus* (*L.*) *brevis* (formerly *Lactobacillus brevis*) TMW (Technische Mikrobiologie Weihenstephan) 1.2112 [37] and *Pediococcus* (*P.*) *claussenii* TMW 2.340 synthesize β-glucan as a capsule around the cells by generating ropy colonies. Both strains carry a plasmid encoded glycosyltransferase-2 gene (*gtf-2*) enabling β-glucan synthesis. A lack of the gene upon plasmid loss (*P. claussenii*) or disruption of the gene by a mobile genetic element (*L. brevis*) leads to the loss of this function, generating non-ropy mutant colonies. Studies about the effects of sourdoughs enriched with bacterial β-glucan including the mutant strains profit from a direct comparison [12,30,38]. In a previous study, we have demonstrated the persistence of beer-spoiling *L. brevis* TMW 1.2112 and *P. claussenii* TMW 2.340 in wheat and rye sourdough fermentation, which contained in situ β-glucan [30]. However, the accurate quantification of β-glucan produced in situ, especially when it is present as a cell-bound network, is still a major challenge.

The aim of the present study was to evaluate two methods (high performance liquid chromatography (HPLC)-based and enzyme-linked immuno-sorbent assay (ELISA)-based for the β-glucan quantification in sourdoughs using the respective *gtf-2* deficient strains as controls. Moreover, various parameters (temperature, inoculum size, providing precursor by cocultivation) potentially influencing the β-glucan content during fermentation were analyzed. With sensory evaluation, the potential acceptance of β-glucan enriched wheat and rye sourdough breads by consumers was investigated.

## 2. Materials and Methods 

### 2.1. Strains and Materials

Five different LAB strains were used in this study: β-glucan-forming wild-type (wt) *L. brevis* TMW 1.2112 and *P. claussenii* TMW 2.340 (isogenic with DSM 14800^T^, and ATCC BAA-344^T^) isolated from breweries. Furthermore, two mutant strains were used, which are derived naturally from the β-glucan-forming wild-type strains. *L. brevis* TMW 1.2320 resulted from a spontaneous transposon insertion (*gtf-2-1148*::IS30) and *P. claussenii* TMW 2.2123 resulted from loss of the *gtf-*2-coding plasmid (∆*gtf-2*) [30,39,40]. The mutants were used as negative control strains since they lack the β-glucan formation and form non-ropy colonies. Furthermore, the α-amylase producing strain *Lactiplantibacillus* (*La*.) *plantarum* TMW 1.2330 [37] was incorporated within sourdough fermentation. The α-amylase activity was tested using the API 50 CHL system and performed according to the instruction manuals. 

The LAB were cultivated in modified Man, Rogosa, and Sharpe medium (mMRS) with pH 6.20 at 30 °C as static cultures. The mMRS medium contained (quantities per liter): 10 g of peptone, 5 g of yeast extract, 5 g of meat extract, 4 g of K_2_HPO_4_, 2.6 g of KH_2_PO_4_·3 H_2_O, 3 g of NH_4_Cl, 1 g of Tween 80, 0.5 g of cysteine-HCl, 10 g of maltose, 5 g of glucose, 5 g of fructose, 0.2 g of MgSO_4_·7 H_2_O, and 0.038 g of MnSO_4_·H_2_O [39,41]. Two different flour types were used for sourdough fermentations: rye flour type 1150 (Roland Mills Nord GmbH & Co. KG, Bremen, Germany) and wheat flour type 550 (Eickernmühle GmbH, Lemgo, Germany).

For β-glucan isolation and purification, a semi-defined medium (pH 5.5) as previously described by Dueñas-Chasco et al. with modifications for improved growth and β-glucan formation was used (quantities per liter): 20 g of maltose, 5 g of casamino acids, 3.5 g of a bacto-yeast nitrogen base (Difco), 3.5 g of yeast carbon base (Difco), 0.05 g of MnSO_4_∙H_2_O, 0.05 g of MgCl_2_∙6 H_2_O, 2 g of K_2_HPO_4_·3 H_2_O, 1 g of KH_2_PO_4_, 5 g of sodium acetate, 0.005 g of adenine sulfate, 0.005 g of guanine, 0.005 g of xanthine, 0.005 g of uracil, 4 g of DL-malic acid, and 1 g of Tween80 [22,42]. The cultivation was performed at 30 °C for 30 h before β-glucan isolation.

### 2.2. Sourdough Fermentation

The conditions for the cultivation of the LAB in mMRS medium and sourdough fermentation were according to those reported by Bockwoldt et al. [30]. Fermentations of rye and wheat sourdoughs with a dough yield (DY) of 200 were performed in triplicates upon variation of the conditions. The fermentation temperature of the wheat and rye doughs was once set to 25 °C and, when raised to 35 °C, compared to the initial publication. Furthermore, wheat and rye doughs were inoculated with LAB solution of 1 and 4 mL (adjusted OD_600nm_ 1) per 200 g dough and fermented at 28 °C. The cocultivation in a 1:1 ratio (2 mL adjusted OD_600nm_ 1 per 200 g dough) with the α-amylase producing *La*. *plantarum* TMW 1.2330, and *L. brevis* TMW 1.2112, or *P. claussenii* TMW 2.340 was performed at 28 °C. The incorporation of the α-amylase positive strain based on the results of the study from Fraunhofer et al., which described an increased maltose availability supported the β-d-glucan production by LAB [22]. Samples for β-glucan quantification were taken every 4 h over 24 h and after 48 and 72 h in the initial experiment to identify the relevant period of β-glucan formation. Consequently, samples were taken after 16, 20, and 24 h from sourdoughs of experiments with changing conditions. 

### 2.3. Cell Count, pH, and Analysis of Sourdough Microbiota 

The colony forming units (CFU) per g of sourdough were analyzed in triplicate before fermentation, and after inoculation of the doughs and at the end of fermentation. Inoculated mMRS agar plates were incubated at 30 °C for 48 h before counting. Species identification of 96 colonies per sourdough triplicate was performed with matrix-assisted laser desorption ionization time of flight mass spectrometry (MALDI-TOF MS) [43]. Ropy colonies are a phenotypical characteristic of β-glucan forming strains (wt). This characteristic was analyzed by using a wooden stick to test if the colonies form ropy strands. The ratio of ropy to non-ropy colonies was determined for 100 colonies per sourdough triplicate after 24 h and the results were verified by the results of the MALDI-TOF MS [30]. Additionally, the pH values of the ripe doughs were measured. 

### 2.4. Sourdough Bread Preparation and Baking

The preparation of sourdough breads was done by using the optimized fermentation temperature and fermentation time analyzed in this study. Wheat sourdoughs with *L. brevis* TMW 1.2112 were fermented at 35 °C and rye sourdoughs at 25 °C while wheat and rye sourdoughs with *P. claussenii* TMW 2.340 were fermented at 25 °C. A 24-h fermentation period was selected for all sourdoughs. Wheat sourdough breads were mixed from 72-g (12%) sourdough, 331.9-g wheat flour, 7.4-g salt, 11.1-g fresh yeast, and 177.5-g tap water. The dry components were mixed, sourdough, water, and yeast were added, and everything was mixed with a hand mixer (575 W Bosch MFQ 4885, Abstatt, Germany) for 5 min, which was followed by 10 min when left at room temperature, kneaded for 10 s, and a further 10 min leaving before distributing 500 g into aluminum trays. The doughs were proofed at 32 °C, 80% humidity for 35 min, and baked at 230 °C for 30 min in the oven (Piccolo, Wachtel GmbH, Hilden, Germany) with an initial 20-s steam injection. The valve was closed for the first 20 min and opened for the last 10 min. For rye sourdough breads, 217.9 g of rye flour, and 6.7 g of salt were mixed, and 244.7 g (40.6%) of fermented sourdough, 6.7 g of fresh yeast, and 134.1 g of tap water were added before everything was mixed with the hand mixer for 5 min, which was followed by 20 min leaving at room temperature. A total of 500 g of the dough was distributed into aluminum trays. The doughs were proofed at 32 °C and 80% humidity for 120 min and baked under the previously described wheat bread conditions. The bread loaves were cooled over night before sensory analysis and sampling.

### 2.5. Sensory Analysis 

Wheat and rye sourdough breads were sliced, and cubes of the crumb with sides of 2–3 cm were coded and presented to an untrained panel (*n* = 14 for wheat breads and *n* = 16 for rye breads) in a randomized order. To determine the acceptance of the breads by consumers, an affective test was performed by evaluating five attributes: moisture, texture (two categories: airiness and texture), acidity, and overall acceptance. The panel analyzed wheat and rye sourdough breads of *L. brevis* TMW 1.2112 and *P. claussenii* TMW 2.340, and the control bread without sourdough. A 5-point Hedonic scale was used for the rating of the attributes from one attribute (dry, compact, chewy, mild, and not tasty) to five attributes (moist, fluffy, crumbly, sour, and tasty).

### 2.6. Isolation and Purification of β-Glucan

Beta-glucan of *L. brevis* TMW 1.2112 was harvested by precipitating the culture supernatant, which was followed by dialysis and freeze-drying of the samples. *L. brevis* TMW 1.2112 was cultivated in a semi-defined medium [42] after cultivation for 30 h at 30 °C. The supernatant was collected by centrifugation at 16,000× *g*, 4 °C for 30 min, and precipitated with ice cold ethanol (three-fold) overnight at 4 °C [18]. The precipitate was collected by centrifugation 10,000× *g*, 4 °C for 15 min, and dissolved in distilled water using glass beads (Ø 2.85–3.45 mm) and a benchtop homogenizer (FastPrep^®^-24 MP, MP Biomedical Inc, Eschwege, Germany). The solution was mixed with a final concentration of 0.5 M perchloric acid for protein precipitation for 5 min on ice, which was followed by centrifugation at 13,000× *g* at 4 °C for 2 min. The resulting supernatant was dialyzed for three days against distilled water changed three times a day, using a dialysis membrane with a cut-off of 50 kDa (SERVA Electrophoresis GmbH, Heidelberg, Germany). After freezing the solution at −80 °C, the samples were freeze-dried (Freezone 2.5, Labconco Corporation, USA). Residues of nitrogen compounds in lyophilized samples were quantified by automated Dumas analysis (MAX N exceed analyzer (Elementar Analysensysteme GmbH, Langenselbold, Germany) by using a calibration with aspartic acid [44]. The isolated and purified samples were used for bacterial β-glucan quantification in wheat and rye doughs.

### 2.7. Quantification of the Bacterial β-Glucan

#### 2.7.1. Quantification by HPLC

Hydrolysis of EPS such as β-glucan in fermented doughs was performed according to Rühmkorf et al. and Ua-Arak et al. with modifications listed in the following [45,46]. The doughs were dissolved in deionized water (1:2 *w*/*v*). This is followed by 30 min centrifugation at 8000× *g* and 10 °C. The supernatant was then mixed with 5% perchloric acid (70%) and hydrolyzed at 100 °C for 7 h. The cooled samples were centrifuged at 10,000× *g*, 10 min, and 10 °C. The supernatant was filtered (0.2 µm nylon filters) and stored overnight at 4 °C. This was followed by a second filtration step. The samples were stored at −20 °C until analysis. The hydrolyzed glucose concentration was analyzed by high performance liquid chromatography (HPLC) using a Rezex™ RPM Pb2+ column (Phenomenex, Aschaffenburg, Germany) with degassed and filtered distilled water as eluent with a flow rate of 0.6 mL/min at 85 °C, and 20-μL injection volume. The column was connected to a refractive index (RI) detector, tempered at 35 °C. The total glucose concentration was calculated into theoretical β-glucan, using a correction factor ($\frac{162}{180}$) converting free glucose to anhydroglucose, as it occurs in β-glucan. Sourdough samples of the wild-type strains able to produce bacterial β-glucan were compared with sourdoughs fermented by the ∆*gtf-2* mutants [47]. 

#### 2.7.2. Quantification by ELISA

Werning et al. developed a competitive enzyme-linked immuno-sorbent assay (ELISA) based on the *Streptococcus* (*S.*) *pneumoniae* serotype 37 antibodies for the quantification of the bacterial β-glucan [48]. In previous studies, these antibodies were used for agglutination tests of β-glucan forming LAB [25,30,49]. In this study, the ELISA assay was used for the quantification of *O*2-substituted (1,3)-β-d-glucan in wheat and rye sourdoughs and baked breads. The β-glucan of *L. brevis* TMW 1.2112 was used for the coating (32.5 µg/mL) of 96-well F8 Maxisorp microtiter plates (Nunc Immuno Module, Thermo Fisher Scientific, Darmstadt, Germany) and the preparation of standards (500, 1000, 5000, 10,000, 50,000, und 100,000 ng/mL) in phosphate buffered saline (PBS) pH of 7.0. The dough samples were dissolved in PBS (1:10 *w*/*v*) using glass beads (Ø 2.85–3.45 mm) and the benchtop homogenizer, which was followed by 10 min centrifugation at 7500× *g* and 4 °C. The supernatant was filtered (0.2-µm nylon filters) and the samples were stored at −20 °C until analysis. The following steps of this assay were performed as previously described by Werning et al. except that the absorbance was measured at 405 nm in a microtiter plate reader (SPECTROstar Nano, BMG Labtech GmbH, Ortenberg, Germany) [48]. Analysis of non-fermented material resulted in very limited cross-reactions. Therefore, non-fermented samples were used as a blank and subtracted from each value.

### 2.8. Statistical Analysis

A one-way ANOVA model combined with the Tukey’s multiple comparisons test (significance level of 0.01) by use of the V. 6.01 GraphPad Prism, GraphPad Software Inc., San Diego, CA, USA, calculated the statistical significance of the β-glucan quantities and the sensory analysis.

## 3. Results

### 3.1. Growth Characteristics of Strains in Wheat and Rye Sourdoughs

The microbiota of the wheat and rye sourdoughs, especially for *L. brevis* TMW 1.2112 (wt), *L. brevis* TMW 1.2320 (*gtf-2-1148*::IS30), *P. claussenii* TMW 2.340 (wt), and *P. claussenii* TMW 2.2123 (*Δgtf-2*), was analyzed by confirming their persistence. Wheat and rye doughs were inoculated with initial cell counts between 10^5^ and 10^6^ CFU/g and reached cell counts between 10^8^ and 10^9^ CFU/g within a 24-h fermentation period. Comparisons of the cell counts between rye and wheat sourdoughs resulted in three-fold to six-fold higher CFUs in rye fermentations with the *L. brevis* strains and two-fold to three-fold higher CFUs with *P. claussenii* strains. MALDI-TOF MS analysis was used to identify inoculated strains at a species level and to confirm their persistence after 24 h of fermentation and revealed ratios with a minimum of 93% except cocultivations. The pH values of the fermented doughs decreased within 16 h between 3.88 and 4.10 and, within 24 h, the pH values ranged from 3.72 ± 0.22 and 3.75 ± 0.17 in wheat and rye sourdoughs, respectively. By increasing the fermentation temperature from 25 °C to 35 °C, the pH values of the doughs decreased more rapidly. Analyses of the strain appearance by MALDI-TOF MS resulted in 98% to 100% during fermentations with different temperatures (25 °C and 35 °C) and dough inoculation sizes with LAB solution (1-mL and 4-mL cell suspension).

#### 3.1.1. Growth Characteristics of Strains Co-Cultivated with *La. plantarum* TMW 1.2330

The microbiota of the cocultivation of β-glucan formers with α-amylase positive *La. plantarum* TMW 1.2330 to provide additional glucose was analyzed after 0 and 24 h. The cocultivation with *La*. *plantarum* TMW 1.2330 resulted in one-fold to two-fold higher total cell counts in rye sourdoughs after 24 h compared with the other approaches. Total cell counts of the wild-type strains were higher when compared to the mutant strains when co-cultivated with *La*. *plantarum* TMW 1.2330. Wheat and rye doughs with *L. brevis* TMW 1.2112 consisted of 45%–48% after 0 h and 26%–48% after 24 h, while *P. claussenii* TMW 2.340 appearance ranged between 50%–66% after 0 h and 61%–70% after 24 h of the respective β-glucan forming strain. The co-cultivation with *La*. *plantarum* TMW 1.2330 resulted in 25% and 48% EPS positive colonies for *L. brevis* TMW 1.2112 and 8% and 5% ropy colonies for *P. claussenii* TMW 2.340 in wheat and rye sourdough, respectively.

#### 3.1.2. Determining Plasmid Stability

The location of the *gtf-2* gene on a plasmid and the ropy phenotype of colonies allows determination of plasmid stability. The ratio of ropy to non-ropy colonies is important regarding the interpretation of quantified β-glucan. In all experiments, *L. brevis* TMW 1.2112 (wt) colonies were ropy at the beginning and the end of wheat and rye sourdough fermentations. In contrast, the ratio of ropy to non-ropy *P. claussenii* TMW 2.340 (wt) colonies was 16% to 95% straight after inoculation (0 h) and 34% to 98% within 24 h, respectively. The mutant strain *L. brevis* TMW 1.2320 (*gtf-2-1148*::IS30) showed no reversion to the wild-type phenotype in any of the experiments and, therefore, the transposon insertion within the *gtf-2* gene was highly stable. Since *P. claussenii* TMW 2.2123 (Δ*gtf-2*) resulted from losing the *gtf-2*-coding plasmid, a reversion to the ropy phenotype was not expected and was not observed (Tables S1 and S2).

### 3.2. Quantification of β-Glucan in Fermented Sourdoughs 

#### 3.2.1. Quantification by HPLC

**Figure 1:**
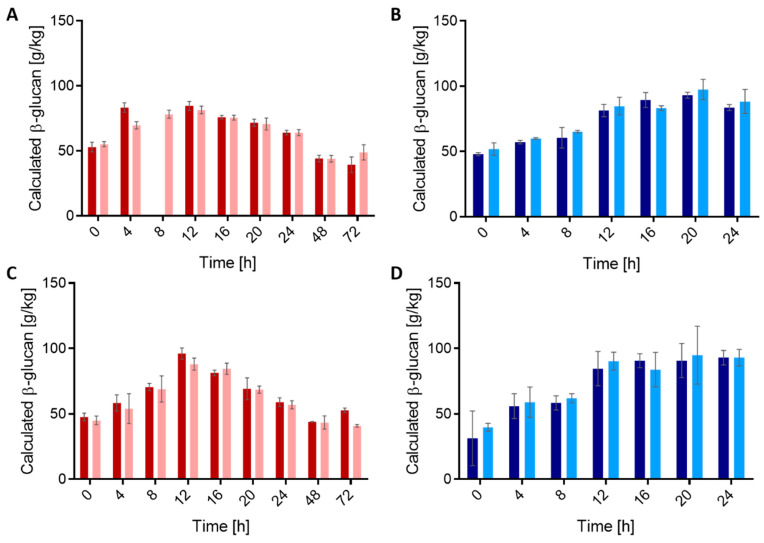
Calculated β-glucan concentration of the total glucose content in wheat and rye sourdoughs measured by HPLC. (**A**) *L. brevis* TMW 1.2112 (▋) and *L. brevis* TMW 1.2320 (▋) in wheat sourdough, (**B**) *P. claussenii* TMW 2.340 (▋) and *P. claussenii* TMW 2.2123 (▋) in wheat sourdough, (**C**) *L. brevis* TMW 1.2112 and *L. brevis* TMW 1.2320 in rye sourdough, and (**D**) *P. claussenii* TMW 2.340 and *P. claussenii* TMW 2.2123 in rye sourdough. The calculated β-glucan amounts were analyzed from biological triplicates with standard deviations.

To monitor the time course of β-glucan formation, the sourdoughs were fermented at 28 °C with sampling every 4 h over 24 h as well as after 48 and 72 h. After 24 h, sampling of *P. claussenii* TMW 2.340 (wt) and *P. claussenii* TMW 2.2123 (Δ*gtf-2*) was terminated. HPLC was used to quantify bacterial β-glucan after perchloric acid hydrolyzation and calculation of the β-glucan concentrations from the total glucose concentration. The wild-type strains and mutants were compared for these differences. The initial calculated β-glucan concentrations of the wheat and rye doughs ranged between 47.9 and 51.8 g/kg as well as 31.3 and 47.5 g/kg, respectively (Figure 1).

Within the first 12 h, the calculated β-glucan concentrations of wheat sourdoughs fermented by *L. brevis* TMW 1.2112 (wt) increased to 84.5 ± 3.4 g/kg and *L. brevis* TMW 1.2320 (*gtf-2-1148*::IS30) to 81.4 ± 3.0 g/kg. A similar trend was detected after 12 h for rye sourdoughs (Figure 1A,C). After another 12 h, the β-glucan concentrations in wheat and rye sourdoughs subsequently decreased close to the initial values. The concentration declined further and, after 72 h, the β-glucan concentration increased again for *L. brevis* TMW 1.2320 (*gtf-2-1148*::IS30) in wheat sourdough and for *L. brevis* TMW 1.2112 (wt) in rye sourdough.

The β-glucan concentration of the wheat and rye sourdoughs with *P. claussenii* TMW 2.340 (wt) and *P. claussenii* TMW 2.2123 (Δ*gtf-2*) increased constantly (Figure 1B,D) during 24 h of fermentation. After 16 h, the β-glucan concentrations in the sourdoughs with the ∆*gtf-2* mutant dropped and were below the concentration values of the wild-type. The maximal β-glucan concentrations of the wild-type was 71.6 ± 1.4 g/kg in wheat sourdoughs after 20 h and 81.3 ± 2.1 g/kg in rye sourdoughs after 16 h. During the 24-h fermentation, similar trends in β-glucan release from all *P. claussenii* sourdoughs were observed.

Changes in calculated β-glucan concentrations of the sourdoughs were almost identical between the same species strains with no significant differences between the wild-type strains, which are able to produce bacterial β-glucan and the ∆*gtf-2* mutants. An actual quantification by comparing calculated β-glucan concentrations of the wt and respective ∆*gtf-2* strain was not effective. 

#### 3.2.2. Quantification by ELISA

**Figure 2:**
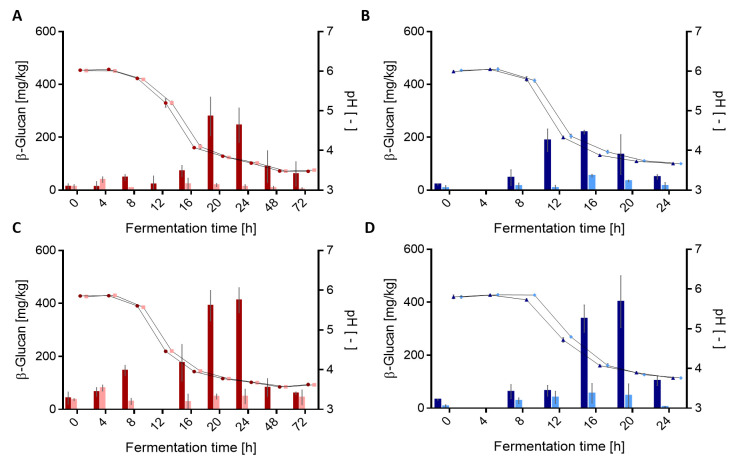
Changes in β-glucan concentration measured by ELISA and pH values during wheat and rye sourdough fermentations. (**A**) *L. brevis* TMW 1.2112 (●) and *L. brevis* TMW 1.2320 (■) in wheat sourdough. (**B**) *P. claussenii* TMW 2.340 (▲) and *P. claussenii* TMW 2.2123 (♦) in wheat sourdough, (**C**) *L. brevis* TMW 1.2112 and *L. brevis* TMW 1.2320 in rye sourdough, and (**D**) *P. claussenii* TMW 2.340 and *P. claussenii* TMW 2.2123 in rye sourdough. The β-glucan amounts and pH values were analyzed by biological triplicates with standard deviations.

In addition to the HPLC analysis, the same samples were analyzed by ELISA to quantify the bacterial β-glucan in wheat and rye sourdoughs. The initial β-glucan concentrations of the wheat and rye doughs were 14.4 ± 9.0 and 21 ± 18.5 mg/kg, respectively (Figure 2).

The β-glucan concentration increased within 20 h to a maximal β-glucan concentration for *L. brevis* TMW 1.2112 (wt) with 279 ± 73.3 mg/kg in wheat sourdoughs. This was significantly higher when compared to *L. brevis* TMW 1.2320 (*gtf-2-1148*::IS30) with 19.1 ± 5.9 mg/kg. After the maximum was reached, a decrease in the β-glucan concentration to 88.7 ± 60.6 mg/kg after 48 h and 61.2 ± 46.7 mg/kg after 72 h (Figure 2A) was detected. The maximal β-glucan concentration in rye sourdoughs fermented by *L. brevis* TMW 1.2112 was measured after 24 h with 412.3 ± 47.4 mg/kg and, therefore, significantly higher when compared to the isogenic β-glucan non-producing *L. brevis* TMW 1.2320 (48.7 ± 27.4 mg/kg). Within a 72-h fermentation period, the β-glucan concentration of *L. brevis* TMW 1.2112 decreased again to 60.8 ± 5.4 mg/kg.

Furthermore, the formation of the bacterial β-glucan by *P. claussenii* TMW 2.340 (wt) was observed and resulted in maximal β-glucan concentrations after 16 h in wheat sourdoughs (219.9 ± 7 mg/kg) and after 20 h in rye sourdough with 402.3 ± 69.4 mg/kg (Figure 2C,D). After the maximal values were reached, the β-glucan concentrations decreased down to ≤110 mg/kg within 24 h of fermentation. The measured β-glucan concentrations of the Δ*gtf-*2 mutants were at a steady low level during wheat and rye sourdough fermentation. During fermentation, the pH values of the wild-type sourdough decreased faster than the respective doughs with the *gtf-*2 deficient mutant until merging after 20 h. Using ELISA, the differences between the wild-type strains able to produce β-glucan and Δ*gtf-*2 mutants were significant. Therefore, this method was used for β-glucan quantification in future experiments.

### 3.3. Parameters Influencing In Situ Formation of β-Glucan in Wheat and Rye Sourdoughs

In the following, the impact of changing several sourdough fermentation parameters to increase the β-glucan formation by LAB were analyzed. The fermentation temperature was decreased to 25 °C and increased to 35 °C. The inoculation amount was halved and doubled and a cocultivation adding *La*. *plantarum* TMW 1.2330 was performed. The previous results of fermentations at 28 °C using 2 mL of cell suspension adjusted to OD_600nm_ 1 per 200 g dough were used as a base for changes in β-glucan concentration and the period for maximum β-glucan production (between 16 and 24 h), which was identified in Section 3.2. The β-glucan produced by *L. brevis* TMW 1.2112 (wt) and *P. claussenii* TMW 2.340 (wt) were quantified by ELISA.

Temperature effects on β-d-glucans production in wheat sourdoughs was observed at 25, 28, and 35 °C (Table 1). An increase of the fermentation temperature up to 35 °C resulted in a significantly higher β-glucan concentration at 24 h with *L. brevis* (506.2 ± 101.6 5 mg/kg) compared to 25 and 28 °C. In contrast, this led to a decrease of the fermentation temperature to 25 °C from a significant maximal concentration (521.1 ± 57.4 mg/kg) after 16 h for *P. claussenii.* It must be considered that, within the temperature experiment, the ratio of ropy *P. claussenii* colonies was 96% and, therefore, the highest in all wheat experiments. Furthermore, with higher fermentation temperatures, the pH values of both strains decreased faster. In a 24-h fermentation period at 25 °C, the pH value was 3.85 ± 0.09 and, at 35 °C, the pH was 3.56 ± 0.06. 

Variations of the inoculation rate with *L. brevis* resulted in similar concentrations compared to the base value (28 °C) except changes in fermentation time and higher maximal concentrations within doubled inoculated doughs. The doubling of the inoculation rate resulted in a 4 h earlier concentration maximum and concentration values of halving the inocula suggested that the maximum is reached. After 24 h, similar trends were observed with *P. claussenii*. In addition, a plasmid loss was observed for 66% and 59% of pediococci cells, respectively. 

Wheat sourdoughs co-fermented by *L. brevis* or *P. claussenii* and the α-amylase producing *La*. *plantarum* TMW 1.2330 resulted in the lowest maximum β-d-glucans concentrations after 24 h. The concentrations ranged between 1.7 ± 5.2 and 43.8 ± 6 mg/kg for *P. claussenii* and *L. brevis*, respectively. Co-cultures of *P. claussenii* with *La*. *plantarum* contained 61% of *P. claussenii* with a ratio of only 8% ropy colonies while co-cultures with *L. brevis* contained 26% *L. brevis* colonies and all were ropy. The remaining percent in both co-cultures was *La*. *plantarum*. These results are more comprehensible as lower numbers of EPS positive cells might have led to lower β-glucan concentrations. The cocultivations in wheat and in rye doughs were generally less productive in β-glucan formation, which might be the result of up to 70% of *P. claussenii* cells with only 5% ropy colonies and 48% of *L. brevis* cells while the remaining percent was *La*. *plantarum*.

Compared to the results of the wheat sourdough fermentations, the β-glucan concentrations in rye sourdoughs were slightly higher (Table 2). The CFUs of both strains were up to six-fold higher in rye fermentation than in wheat fermentation. *L. brevis* reached its maximum β-glucan concentration of 573.6 ± 78.7 mg/kg in rye sourdoughs when fermented at 25 °C for 24 h. Within 20 to 24 h, *P. claussenii* maximum was ≤624.7 ± 62.8 mg/kg at 25 °C with a ratio of ropy colonies of 98%, which was the maximum within all rye fermentations. 

The pH values were again lower with a higher fermentation temperature: 3.66 ± 0.08 (35 °C) and 3.85 ± 0.06 (25 °C). The decrease of the pH values seemed not to influence the β-glucan formation. Increasing the inoculum resulted in similar trends, as observed during wheat fermentation (lower initial cell numbers and lower β-glucan concentrations).

**Figure 3:**
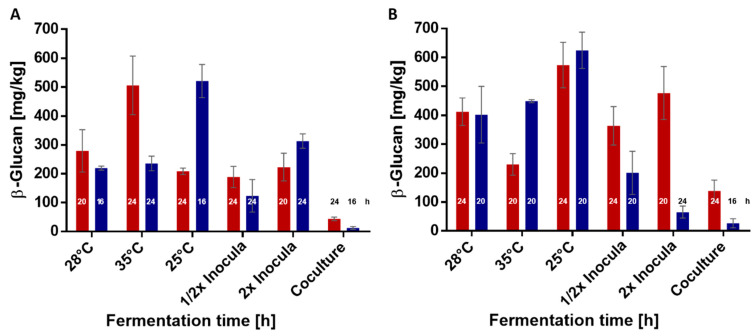
Maximal β-glucan concentrations in fermented wheat and rye sourdoughs under different conditions. (**A**) *L. brevis* TMW 1.2112 (▋) and *P. claussenii* TMW 2.340 (▋) in wheat sourdough and (**B**) *L. brevis* TMW 1.2112 and *P. claussenii* TMW 2.340 in rye sourdough with the respective fermentation times in h. Values are means of biological triplicates including standard deviations.

Figure 3 shows the maximal β-glucan concentrations with different fermentation conditions (temperature, inoculation size, and co-cultivation). Generally, the maximal β-glucan concentration was reached earlier with *P. claussenii* than with *L. brevis.* In wheat sourdough fermentations, the fermentation temperature had the most significant influence for β-glucan formation by *L. brevis* TMW 1.2112 and *P. claussenii* TMW 2.340. While *L. brevis* seemed to prefer 35 °C for increased β-glucan formation, *P. claussenii* preferred 25 °C (Figure 3A). The measured β-glucan concentration in rye sourdoughs were, in general, higher when compared to wheat sourdoughs, which might be attributed to higher cell counts. Changes in the fermentation temperature and amount of inoculum influenced the β-glucan formation in rye sourdoughs the most. However, these effects were interfered with random extensive loss of the ability to form EPS by *P. claussenii*. On the contrary, the EPS formation of *L. brevis* TMW 1.2112 colonies were positive in all experiments. Both strains formed the highest β-glucan levels in rye doughs when fermented at 25 °C.

### 3.4. Analysis of β-Glucan-Enriched Bread Characterisics

**Figure 4:**
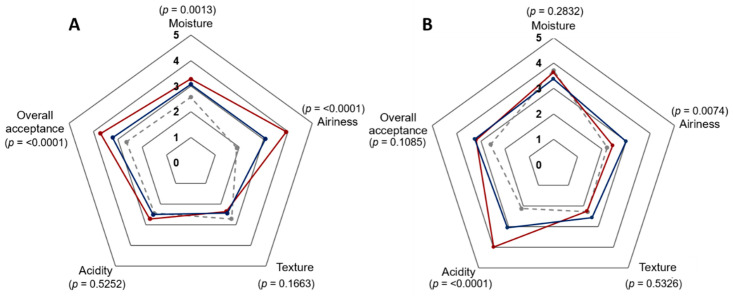
Consumer acceptability of β-glucan enriched wheat and rye breads. (**A**) Wheat bread with *L. brevis* TMW 1.2112 (red), *P. claussenii* TMW 2.340 (blue) compared to the control (dashed line, grey). (**B**) Rye bread with *L. brevis* TMW 1.2112, *P. claussenii* TMW 2.340 compared to the control. Data are presented in a 5-point hedonic scale. Sensory attributes with *p* ≤ 0.01 indicate a significant difference among the samples.

Sensory analysis was performed, using an affective test on wheat and rye sourdough breads of *L. brevis* TMW 1.2112 and *P. claussenii* TMW 2.340 compared to the control. The untrained panel used a 5-points Hedonic scale to rate five attributes (moisture, airiness, texture, acidity, and overall acceptance) of the breads. Mean values of the results are presented using spider diagrams including statistical significance values (Figure 4). Significant differences in the sensory quality of the wheat sourdough breads, especially for the moisture, airiness, and overall acceptance were observed. Wheat sourdough breads of *L. brevis* (*p* < 0.0001) and *P. claussenii* (*p* = 0.0001) were significantly fluffier than the control, while the bread of *L. brevis* was significantly moister (*p* = 0.0022) than *P. claussenii* (*p* = 0.0266) and the control breads. Within the two categories of texture and acidity, the panel detected no significant differences between the three samples. In the category of overall acceptance, the panel had significantly higher preference for the wheat breads of *L. brevis* with an average of 3.7 (*p* < 0.0001) and 3.2 for *P. claussenii* (*p* = 0.0027) than the control with an average value of 2.6 (dislike slightly). 

In addition, the same sensory analysis was performed with rye sourdough breads (Figure 4B). The acidity of the breads resulted in high ratings of *L. brevis* with an average of four compared to *P. claussenii* (3) and the control bread (2.1). The statistical analysis resulted in significant differences between the three samples with *p* < 0.0001 for *L. brevis* and the control, *p* = 0.0080 for *L. brevis* and *P. claussenii*, and *p* = 0.0019 for *P. claussenii* and the control bread. The airiness of *P. claussenii* rye sourdough breads were rated with 3 while *L. brevis* was rated with 2.4 and the control bread was rated with 2.2. Despite the higher rated acidity and the lower rated fluffiness, the overall acceptance of *L. brevis* was rated with 3.2 and, similarly to the rating of *P. claussenii*, it was rated with 3.3. Within the attributes of moisture, texture, and overall acceptance, no significant differences were observed between the samples.

## 4. Discussion

In the present study, we provide insights into the formation of β-d-glucan produced by LAB during wheat and rye sourdough fermentation. The competitiveness of two brewery isolates *L. brevis* and *P. claussenii* in sourdough fermentations against the endogenous microbiota was demonstrated in our previous study [30]. In the same study, a significant effect of β-glucan-forming *L. brevis* TMW 1.2112 on the viscosity of wheat sourdoughs compared to *P. claussenii* TMW 2.340 and the mutants was described but missed quantity information.

A common method to quantify β-glucans is the determination of glucose by HPLC after acidic hydrolysis of the β-glucan with perchloric acid. Ua-Arak et al. successfully used a similar method for the quantification of fructose monomers of in situ produced levan in buckwheat sourdoughs [46]. However, in addition to bacterial β-glucan, further polysaccharides, e.g., starch and cereal β-glucan, are sources, which release glucose upon acid hydrolysis [50]. The β-glucan calculation of hydrolyzed samples inevitably leads to an overestimation of the β-glucan concentration. In this study, HPLC analysis revealed that differences between β-glucan producing wt strains and non-producer strains were not significant and gave contradictory results with higher calculated β-glucan concentrations with non-producers than with EPS-forming wild-type strains. As the comparison of the β-glucan concentration between the wild-types and mutants was crucial for a quantification by HPLC, this method might not be specific enough. The initial increase of glucose (later calculated β-glucan) observed could be explained by the phosphorolytic cleavage of the preferred maltose by maltose phosphorylase (MP) of *L. brevis* [51]. Within the genome sequence of *L. brevis* TMW 1.2112, a putative MP (AZI09_01010) was detected while no such genes were found for *P. claussenii* TMW 2.340 [39,52]. 

The formation of the type 37 capsule of *S*. *pneumoniae* is driven by the single gene *tts*, which shows similar sequence characteristics of other β-glycosyltransferase genes as the *gtf-2* gene of *L. brevis* TMW 1.2112 and *P. claussenii* TMW 2.340 [53]. Due to this similarity, the quantification of the β-glucan using serotype 37 antibodies was possible. In our study, the β-glucan quantification by ELISA applied to β-glucan produced during growth in medium demonstrated a high sensitivity by detecting even slight traces in the doughs. In contrast to barley, oat, and rice fermentations, cross-reactions between the antibodies with wheat and rye samples were observed, and, therefore, non-fermented material was used as a blank and subtracted from fermented material [12]. Nevertheless, even in samples with the *gtf-2* deficient strains, β-glucan concentrations up to 80 mg/kg sourdough were measured after blank correction. Fermented sourdoughs contain yeast β-glucan (mixed linked (1,3) and (1,6)-β-d-glucan) and other polysaccharides, which potentially cross-react with the assay [54,55,56,57]. Werning et al. analyzed the binding of the antibodies to curdlan (linear (1,3)-β-d-glucan), laminarin (6-substituted (1,3)-β-d-glucan), and xanthan a HePS [48]. While xanthan was unable to bind and curdlan had only a weak affinity, laminarin was able to compete almost equally with the *O*2-substituted (1,3)-β-d-glucan [48]. However, since laminarin is a marine-based polysaccharide from algae, in our study, this aspect was insignificant [58]. Despite these minor drawbacks, the immunological assay proved to be a suitable quantification method of β-glucan in cereal matrices since significant increases of produced β-glucan followed by decreases were observable. Within 20 to 24 h, the β-glucan concentrations of rye sourdoughs fermented by *L. brevis* TMW 1.2112 and *P. claussenii* TMW 2.340 increased up to 412 mg/kg and 402 mg/kg, respectively, and corresponded with the results of the oat flour fermentation by *Pediococcus* (*P.*) *parvulus* 2.6, which produced 139.7 ± 40.8 mg/L β-d-glucan within 24 h [12].

Since the artificial addition of EPS in doughs is less effective as in situ-formed EPS, an increase of β-d-glucan formation by LAB in sourdoughs was tested by changing the fermentation conditions [23,59]. One approach was the addition of α-amylase producing *La*. *plantarum* TMW 1.2330 to increase available glucose and maltose by degrading starch to promote growth and production of β-d-glucan by *L. brevis* TMW 1.2112 or *P. claussenii* TMW 2.340. However, it resulted in the lowest β-d-glucan amounts of this study. Notably, under these competitive conditions, lower cell counts of the β-d-glucan mutant strains compared to the wild-type strains were observed (Table S1 and Table S2). *P. claussenii* TMW 2.2123 was almost displaced by *La*. *plantarum*. EPS encapsulated cells might be better protected from inhibitory compounds as more vulnerable mutant strains [60]. Finally, the starch degradation was not sufficient for increasing the β-d-glucan formation and compensating substrate utilization by *La*. *plantarum* at the same time. The addition of 10% maltose to wheat and rye sourdoughs fermented by *L. brevis* TMW 1.2112 did not lead to increased β-d-glucan, and unveiled the β-d-glucan formation is not restricted by the constituents in flour and dough systems (unpublished data). Furthermore, the high cell counts (10^8^ to 10^9^ CFU/g), characteristically for traditional fermented sourdoughs, excluded substrate limitation [61].

Sourdoughs inoculated with different amounts of cells featured similar cell counts and pH values at the end of fermentation, but the course of β-d-glucan formation differed as higher inoculated doughs (≤6.6 × 10^6^ CFU/g) reached earlier and higher maximum glucan concentrations. While lower inoculated doughs (≤1.6 × 10^6^ CFU/g) seemed to reach their maximum at a later stage in fermentation. Thus, β-d-glucan concentrations in dough fermentation seems not to be linked to the cell counts of the producer cells.

In our previous study, we described the loss of EPS formation by *P. claussenii* TMW 2.340 while formation in *L. brevis* TMW 1.2112 was perfectly stable [30]. The same effects were demonstrated in this study. The β-glycosyltransferases genes are plasmid-encoded traits in both strains. Plasmid instability and the resulting loss of β-d-glucan formation might be prevented by a toxin/antitoxin-system in *L. brevis* but not in *P. claussenii* [30,62,63]. Additionally, the relatively high β-d-glucan concentration at high cell counts with low percentages of EPS-forming colonies may be explained by an additional regulation of the plasmid copy number, regulation of the amount of enzyme at a transcriptional level, or the regulation of the enzyme activity [64,65,66]. 

The most significant impact on β-d-glucan formation was observed by changing the fermentation temperature (Table 1 and Table 2). This approach obtained concentrations of 624.7 ± 62.8 mg/kg in rye and 521.1 ± 57.4 mg/kg in wheat sourdoughs fermented with *P. claussenii* TMW 2.340 at an optimal temperature of 25 °C. The comparably highly stable β-d-glucan formation in *P. claussenii* TMW 2.340, with 95% to 99% EPS-forming colonies could be a reason for such high concentrations. Furthermore, the preferred sourdough fermentation temperature of *P. claussenii* seemed to be 25 °C, and the previous 28 °C might have been unfavorable for this strain. *L. brevis* produced the highest β-d-glucan concentrations at 35 °C and 25 °C in wheat and rye sourdoughs, respectively. The selected fermentation temperatures (25 and 35 °C) corresponded with the optimum growth temperature ranges of *P. claussenii* and *L. brevis* sp. [67,68,69]. Upon cultivation in media of *L. brevis* TMW 1.2112, Fraunhofer reported that β-d-glucan formation was increased at lower pH values [25]. This impact was not observed for sourdough fermentations. Instead, the opposite occurred, as more β-d-glucan was quantified upon a slower pH decrease. 

Pérez-Ramos et al. used *P. parvulus* 2.6 to ferment oats and produced 659.4 ± 45.18 mg/L β-d-glucan within 64 h. A decreasing trend of the β-d-glucan concentration as observed in our study was not described. The oat matrices were initially fermented by *La. plantarum* and heated before adding *P. parvulus* 2.6 [12,70]. The pre-fermentation may have accelerated starch breakdown, released soluble monosaccharides and disaccharides, which can be used as a substrate from *P. parvulus* 2.6 while the heating inactivated largely cereal microbiota and endogenous enzymes able to degrade formed bacterial β-d-glucan [57,71]. In contrast, in our study, raw materials were used and bacterial β-d-glucan degradation could be expedited by cereal-based enzyme activities, e.g., endo-β-glucanase [71]. In addition, cell-own glycosyl hydrolases might decrease the β-glucan amount, e.g., putative endoglucanase (AZI09_02135) or 1,3-β-d-glucanase (AZI09_02170) of *L. brevis* TMW 1.2112 to maintain the substrate supply after depletion of environmental sources [25,72,73,74]. Since the bacterial β-glucan is mainly formed as capsular EPS, a relatively large fraction of the EPS gets lost during sample preparation for ELISA due to centrifugation and filtration steps [30,38]. Therefore, it can be reasonably assumed that the concentration of the β-d-glucan is much higher within the fermented wheat and rye sourdoughs. 

The sensory quality of wheat and rye sourdough breads fermented with *L. brevis* TMW 1.2112 and *P. claussenii* TMW 2.340 was analyzed by an untrained panel. Wheat bread fermented with *L. brevis* was, overall, higher scored than wheat bread with *P. claussenii.* Whereas rye bread with *L. brevis* was graded predominantly sour and *P. claussenii* not. Nevertheless, the overall acceptance of both rye breads was similar and supposedly is related to the unique flavor of sourdough breads produced by heterofermentative LAB compared to homofermentative LAB. Banu et al. described that a trained panel rated the overall flavor intensity of rye bread crumbs of heterofermentative LAB (including *L. brevis sp.*) higher as homofermentative produced rye breads [75,76]. In our previous study, we demonstrated that homofermentative *P. claussenii* produced only lactic acid while heterofermentative *L. brevis* produced lactic and acetic acid during wheat and rye sourdough fermentation [30]. Acetic acid can improve bread quality, such as the lactate and acetate ratio contributing to the flavor [1,77,78]. The sensory analysis demonstrated the overall acceptance of sourdough breads fermented by *L. brevis* or *P. claussenii* and resulted in a significant contribution to bread quality.

## 5. Conclusions

Taken together, this study compares homofermentative *P. claussenii* and heterofermentative *L. brevis* in β-glucan formation during wheat and rye sourdough fermentation. In our hands, quantification of glucan with an ELISA approach was superior to the indirect determination of deliberated glucose after hydrolysis. It was demonstrated that the fermentation process can significantly influence the β-d-glucan concentration of the sourdoughs. The cell count and EPS level seemed to be independent of each other, implying external factors trigger the EPS production. Temperature control proved to mainly influence EPS formation depending on flour type and microbiota. Since high EPS concentrations are favorable with respect to techno-functional properties and health beneficial effects, an increase of the EPS amount is an important factor. This applies in particular to HePS and β-d-glucans, which are formed in low quantities compared to HoPS. However, the β-d-glucan concentration declined during fermentation and, therefore, further investigation should be performed to determine the factors responsible for the decrease of the β-d-glucan during fermentation, which is most likely cereal and cell-own glycosyl hydrolases. Compared with sucrose-fed LAB for in situ HoPS formation, the β-d-glucan forming LAB can be used for clean label products without residual sweetness or excessive acetic acid formation by utilizing naturally contained monosaccharides and disaccharides of the flour for optimum growth. 

## Figures and Tables

**Table 1:** β-glucan [mg/kg] production by varying parameters in wheat fermentation by *L. brevis* TMW 1.2112 and *P. claussenii* TMW 2.340.

Strain	Temperature	Inoculum Size	Fermentation Time [h]		
			16	20	24
*L. brevis* TMW 1.2112	25 °C	1×	35 ± 29.7	104.5 ± 15	208.5 ± 11.2
	28 °C	1×	72.2 ± 21.5	279 ± 73.3	245.9 ± 65
	35 °C	1×	131.1 ± 43.7	91.6 ± 48.7	506.2 ± 101.6
	28 °C	1/2×	60.6 ± 6	172.3 ± 12.6	188.7 ± 36.5
	28 °C	2×	21.1 ± 2.2	223.3 ± 47.7	111.1 ± 10.1
	28 °C	1/2× *L. brevis* 1/2× *La*. *plantarum*	46.5 ± 8.2	14.1 ± 3.5	43.8 ± 6
*P. claussenii* TMW 2.340	25 °C	1×	521.1 ± 57.4	222 ± 52.8	220.4 ±28.2
	28 °C	1×	219.9 ± 7	134.5 ± 75.4	50.4 ± 8.5
	35 °C	1×	142.8 ± 18.	224.9 ± 32.9	235.6 ± 25.4
	28 °C	1/2×	53.5 ± 27.9	64.5 ± 36.2	123.5 ± 56.4
	28 °C	2×	218.9 ± 31.1	178.4 ± 13.7	313.2 ± 24.9
	28 °C	1/2× *P. claussenii* 1/2× *La*. *plantarum*	12.5 ± 4.9	11.4 ± 11.9	1.7 ± 5.2

**Table 2:** β-glucan [mg/kg] production by varying parameters in rye fermentation by *L. brevis* TMW 1.2112 and *P. claussenii* TMW 2.340.

Strain	Temperature	Inoculum Size	Fermentation Time [h]		
			16	20	24
*L. brevis* TMW 1.2112	25 °C	1×	399.1 ± 39.2	422.4 ±56.4	573.6 ± 78.7
	28 °C	1×	175.9 ± 69.4	391.7 ± 57.5	412.3 ± 47.4
	35 °C	1×	195.7 ± 40.9	230 ± 37.5	220.6 ±52.9
	28 °C	1/2×	191.3 ± 27.2	314.4 ± 34.6	363.6 ± 66.4
	28 °C	2×	41.2 ± 10.7	477 ± + 91.7	468.7 ± 90.2
	28 °C	1/2× *L. brevis* 1/2× *La*. *plantarum*	111.4 ± 8.8	99.3 ± 38.1	138.7 ± 37.1
*P. claussenii* TMW 2.340	25 °C	1×	493.6 ± 52.7	624.7 ± 62.8	623 ± 11.4
	28 °C	1×	338.5 ± 51.5	402.2 ± 98.1	104 ± 18.1
	35 °C	1×	346 ± 36.3	514.1 ± 17.8	448.7 ± 5.2
	28 °C	1/2×	146.3 ± 78.1	200.8 ± 74.8	195.2 ± 121.1
	28 °C	2×	400.4 ± 117.3	328.8 ±71.9	429.5 ±129.8
	28 °C	1/2× *P. claussenii* 1/2× *La*. *plantarum*	29.8 ± 10.5	19.2 ± 21.1	26.4 ± 15.7

## Data Availability

Data sharing not applicable.

## Supplementary Materials

The following are available online at https://www.mdpi.com/2304-8158/10/3/547/s1. Table S1: Results of wheat sourdough analyses: cell count, pH values, MALDI-TOF MS, and ratio of EPS positive colonies and Table S2: Results of rye sourdough analyses: cell count, pH values, MALDI-TOF MS, and ratio of EPS positive colonies.

## References

[1] Leroy F., De Vuyst L. (2004). Lactic acid bacteria as functional starter cultures for the food fermentation industry. Trends Food Sci. Tech..

[2] Ray B. (2019). Food Biopreservatives of Microbial Origin.

[3] Katina K., Heiniö R.-L., Autio K., Poutanen K. (2006). Optimization of sourdough process for improved sensory profile and texture of wheat bread. LWT Food Sci. Technol..

[4] Arendt E.K., Ryan L.A., Dal Bello F. (2007). Impact of sourdough on the texture of bread. Food Microbiol..

[5] Gerez C.L., Torino M.I., Rollán G., de Valdez G.F. (2009). Prevention of bread mould spoilage by using lactic acid bacteria with antifungal properties. Food Control..

[6] Galle S., Arendt E.K. (2014). Exopolysaccharides from sourdough lactic acid bacteria. Crit. Rev. Food Sci. Nutr..

[7] Rühmkorf C., Rübsam H., Becker T., Bork C., Voiges K., Mischnick P., Brandt M.J., Vogel R.F. (2012). Effect of structurally different microbial homoexopolysaccharides on the quality of gluten-free bread. Eur. Food Res. Technol..

[8] Chen X.Y., Levy C., Ganzle M.G. (2016). Structure-function relationships of bacterial and enzymatically produced reuterans and dextran in sourdough bread baking application. Int. J. Food Microbiol..

[9] Galle S., Schwab C., Arendt E.K., Ganzle M.G. (2011). Structural and rheological characterisation of heteropolysaccharides produced by lactic acid bacteria in wheat and sorghum sourdough. Food Microbiol..

[10] Poutanen K., Flander L., Katina K. (2009). Sourdough and cereal fermentation in a nutritional perspective. Food Microbiol..

[11] Russo P., Lopez P., Capozzi V., de Palencia P.F., Duenas M.T., Spano G., Fiocco D. (2012). Beta-glucans improve growth, viability and colonization of probiotic microorganisms. Int. J. Mol. Sci..

[12] Pérez-Ramos A., Mohedano M.L., López P., Spano G., Fiocco D., Russo P., Capozzi V. (2017). In situ β-glucan fortification of cereal-based matrices by Pediococcus parvulus 2.6: Technological aspects and prebiotic potential. Int. J. Mol. Sci..

[13] Salazar N., Gueimonde M., De Los Reyes-Gavilán C.G., Ruas-Madiedo P. (2016). Exopolysaccharides produced by lactic acid bacteria and bifidobacteria as fermentable substrates by the intestinal microbiota. Crit. Rev. Food Sci. Nutr..

[14] Welman A.D., Maddox I.S. (2003). Exopolysaccharides from lactic acid bacteria: Perspectives and challenges. Trends Biotechnol..

[15] Liu C.F., Tseng K.C., Chiang S.S., Lee B.H., Hsu W.H., Pan T.M. (2011). Immunomodulatory and antioxidant potential of *Lactobacillus exopolysaccharides*. J. Sci. Food Agric..

[16] Laiño J., Villena J., Kanmani P., Kitazawa H. (2016). Immunoregulatory effects triggered by lactic acid bacteria exopolysaccharides: New insights into molecular interactions with host cells. Microorganisms.

[17] Saadat Y.R., Khosroushahi A.Y., Gargari B.P. (2019). A comprehensive review of anticancer, immunomodulatory and health beneficial effects of the lactic acid bacteria exopolysaccharides. Carbohydr. Polym..

[18] Notararigo S., Nacher-Vazquez M., Ibarburu I., Werning M.L., de Palencia P.F., Duenas M.T., Aznar R., Lopez P., Prieto A. (2013). Comparative analysis of production and purification of homo- and hetero-polysaccharides produced by lactic acid bacteria. Carbohydr. Polym..

[19] Galle S., Schwab C., Arendt E., Ganzle M. (2010). Exopolysaccharide-forming *Weissella* strains as starter cultures for sorghum and wheat sourdoughs. J. Agric. Food Chem..

[20] van Hijum S.A., Kralj S., Ozimek L.K., Dijkhuizen L., van Geel-Schutten I.G. (2006). Structure-function relationships of glucansucrase and fructansucrase enzymes from lactic acid bacteria. Microbiol. Mol. Biol. Rev..

[21] De Vuyst L., De Vin F., Vaningelgem F., Degeest B. (2001). Recent developments in the biosynthesis and applications of heteropolysaccharides from lactic acid bacteria. Int. Dairy J..

[22] Fraunhofer M.E., Jakob F., Vogel R.F. (2018). Influence of Different Sugars and Initial pH on β-Glucan Formation by *Lactobacillus brevis* TMW 1.2112. Curr. Microbiol..

[23] Tieking M., Korakli M., Ehrmann M.A., Gänzle M.G., Vogel R.F. (2003). In situ production of exopolysaccharides during sourdough fermentation by cereal and intestinal isolates of lactic acid bacteria. Appl. Environ. Microbiol..

[24] De Vuyst L., Degeest B. (1999). Heteropolysaccharides from lactic acid bacteria. FEMS Microbiol. Rev..

[25] Fraunhofer M. (2018). Characterization of EPS-Producing Brewery-Associated Lactobacilli. Ph.D. Thesis.

[26] Patel S., Majumder A., Goyal A. (2012). Potentials of exopolysaccharides from lactic Acid bacteria. Indian J. Microbiol..

[27] Gobbetti M. (1998). The sourdough microflora: Interactions of lactic acid bacteria and yeasts. Trends Food Sci. Technol..

[28] KučeroVá J. (2009). Effects of location and year on technological quality and pentosan content in rye. Czech J. Food Sci..

[29] Martínez-Anaya M.A. (1996). Enzymes and bread flavor. J. Agric. Food Chem..

[30] Bockwoldt J.A., Stahl L., Ehrmann M.A., Vogel R.F., Jakob F. (2020). Persistence and β-glucan formation of beer-spoiling lactic acid bacteria in wheat and rye sourdoughs. Food Microbiol..

[31] Korakli M., Rossmann A., Ganzle M.G., Vogel R.F. (2001). Sucrose metabolism and exopolysaccharide production in wheat and rye sourdoughs by *Lactobacillus sanfranciscensis*. J. Agric. Food Chem..

[32] Korakli M., Vogel R.F. (2006). Structure/function relationship of homopolysaccharide producing glycansucrases and therapeutic potential of their synthesised glycans. Appl. Microbiol. Biotechnol..

[33] Bounaix M.S., Gabriel V., Morel S., Robert H., Rabier P., Remaud-Simeon M., Gabriel B., Fontagne-Faucher C. (2009). Biodiversity of exopolysaccharides produced from sucrose by sourdough lactic acid bacteria. J. Agric. Food Chem..

[34] Kaditzky S., Seitter M., Hertel C., Vogel R.F. (2007). Performance of *Lactobacillus sanfranciscensis* TMW 1.392 and its levansucrase deletion mutant in wheat dough and comparison of their impact on bread quality. Eur. Food Res. Technol..

[35] Mårtensson O., Chasco M.-D., Irastorza A., Holst O., Rudling M., Norin E., Midtvedt T., Öste R. (2002). Effects of fermented, ropy, non-dairy, oat-based products on serum lipids and the faecal excretion of cholesterol and short chain fatty acids in germfree and conventional rats. Nutr. Res..

[36] Notararigo S., de Las Casas-Engel M., de Palencia P.F., Corbi A.L., Lopez P. (2014). Immunomodulation of human macrophages and myeloid cells by 2-substituted (1-3)-β-D-glucan from *P. parvulus* 2.6. Carbohydr. Polym..

[37] Zheng J., Wittouck S., Salvetti E., Franz C., Harris H.M.B., Mattarelli P., O’Toole P.W., Pot B., Vandamme P., Walter J. (2020). A taxonomic note on the genus *Lactobacillus*: Description of 23 novel genera, emended description of the genus *Lactobacillus* Beijerinck 1901, and union of *Lactobacillaceae* and *Leuconostocaceae*. Int. J. Syst. Evol. Microbiol..

[38] Fraunhofer M.E., Geissler A.J., Wefers D., Bunzel M., Jakob F., Vogel R.F. (2018). Characterization of β-glucan formation by *Lactobacillus brevis* TMW 1.2112 isolated from slimy spoiled beer. Int. J. Biol. Macromol..

[39] Fraunhofer M.E., Geissler A.J., Jakob F., Vogel R.F. (2017). Multiple Genome Sequences of Exopolysaccharide-Producing, Brewery-Associated *Lactobacillus brevis* Strains. Genome Announc..

[40] Suzuki K. (2011). 125th Anniversary Review: Microbiological Instability of Beer Caused by Spoilage Bacteria. J. Inst. Brew..

[41] Schurr B.C., Behr J., Vogel R.F. (2013). Role of the GAD system in hop tolerance of *Lactobacillus brevis*. Eur. Food Res. Technol..

[42] Duenas-Chasco M.T., Rodriguez-Carvajal M.A., Mateo P.T., Franco-Rodriguez G., Espartero J.L., Irastorza-Iribas A., Gil-Serrano A.M. (1997). Structural analysis of the exopolysaccharide produced by *Pediococcus damnosus* 2.6. Carbohydr. Res..

[43] Kern C.C., Usbeck J.C., Vogel R.F., Behr J. (2013). Optimization of Matrix-Assisted-Laser-Desorption-Ionization-Time-Of-Flight Mass Spectrometry for the identification of bacterial contaminants in beverages. J. Microbiol. Methods.

[44] Kupetz M., Geißinger C., Gastl M., Becker T. (2018). Comparison of Dumas and Kjeldahl method for nitrogen determination in malt, wort and beer. Brew. Sci..

[45] Rühmkorf C., Jungkunz S., Wagner M., Vogel R.F. (2012). Optimization of homoexopolysaccharide formation by lactobacilli in gluten-free sourdoughs. Food Microbiol..

[46] Ua-Arak T., Jakob F., Vogel R.F. (2017). Influence of levan-producing acetic acid bacteria on buckwheat-sourdough breads. Food Microbiol..

[47] McCleary B.V., Codd R. (1991). Measurement of (1→3),(1→4)-β-D-glucan in barley and oats: A streamlined enzymic procedure. J. Sci. Food Agric..

[48] Werning M.L., Perez-Ramos A., de Palencia P.F., Mohedano M.L., Duenas M.T., Prieto A., Lopez P. (2014). A specific immunological method to detect and quantify bacterial 2-substituted (1,3)-β-D-glucan. Carbohydr. Polym..

[49] Walling E., Gindreau E., Lonvaud-Funel A. (2005). A putative glucan synthase gene *dps* detected in exopolysaccharide-producing *Pediococcus damnosus* and *Oenococcus oeni* strains isolated from wine and cider. Int. J. Food Microbiol..

[50] Comino P., Shelat K., Collins H., Lahnstein J., Gidley M.J. (2013). Separation and purification of soluble polymers and cell wall fractions from wheat, rye and hull less barley endosperm flours for structure-nutrition studies. J. Agric. Food Chem..

[51] Stolz P., Böcker G., Vogel R.F., Hammes W.P. (1993). Utilisation of maltose and glucose by lactobacilli isolated from sourdough. FEMS Microbiol. Lett..

[52] Pittet V., Abegunde T., Marfleet T., Haakensen M., Morrow K., Jayaprakash T., Schroeder K., Trost B., Byrns S., Bergsveinson J. (2012). Complete genome sequence of the beer spoilage organism *Pediococcus claussenii* ATCC BAA-344T. J. Bacteriol..

[53] Llull D., Munoz R., Lopez R., Garcia E. (1999). A single gene (*tts*) located outside the cap locus directs the formation of *Streptococcus pneumoniae* type 37 capsular polysaccharide: Type 37 pneumococci are natural, genetically binary strains. J. Exp. Med..

[54] Stier H., Ebbeskotte V., Gruenwald J. (2014). Immune-modulatory effects of dietary Yeast β-1,3/1,6-D-glucan. Nutr. J..

[55] Gardiner T., Carter G. (2000). β-glucan biological activities: A review (condensed version). Glyco. Sci. Nutr..

[56] Boone C., Sommer S.S., Hensel A., Bussey H. (1990). Yeast KRE genes provide evidence for a pathway of cell wall beta-glucan assembly. J. Cell Biol..

[57] Andersson A.A., Armö E., Grangeon E., Fredriksson H., Andersson R., Åman P. (2004). Molecular weight and structure units of (1→3, 1→4)-β-glucans in dough and bread made from hull-less barley milling fractions. J. Cereal Sci..

[58] Becker S., Tebben J., Coffinet S., Wiltshire K., Iversen M.H., Harder T., Hinrichs K.U., Hehemann J.H. (2020). Laminarin is a major molecule in the marine carbon cycle. Proc. Natl. Acad. Sci. USA.

[59] Brandt M., Roth K., Hammes W. (2003). Effect of an Exopolysaccharide Produced by *Lactobacillus sanfranciscensis* LTH1729 on Dough and Bread Quality. Sourdough from Fundamentals to Applications.

[60] Sutherland I.W. (2001). Biofilm exopolysaccharides: A strong and sticky framework. Microbiology.

[61] De Vuyst L., Neysens P. (2005). The sourdough microflora: Biodiversity and metabolic interactions. Trends Food Sci. Technol..

[62] Christensen S.K., Mikkelsen M., Pedersen K., Gerdes K. (2001). RelE, a global inhibitor of translation, is activated during nutritional stress. Proc. Natl. Acad. Sci. USA.

[63] Gerdes K., Christensen S.K., Lobner-Olesen A. (2005). Prokaryotic toxin-antitoxin stress response loci. Nat. Rev. Microbiol..

[64] Degeest B., De Vuyst L. (2000). Correlation of Activities of the Enzymes α-Phosphoglucomutase, UDP-Galactose 4-Epimerase, and UDP-Glucose Pyrophosphorylase with Exopolysaccharide Biosynthesis by *Streptococcus thermophilus* LY03. Appl. Environ. Microbiol..

[65] Escalante A., Wacher-Rodarte C., Garcia-Garibay M., Farres A. (1998). Enzymes involved in carbohydrate metabolism and their role on exopolysaccharide production in Streptococcus thermophilus. J. Appl. Microbiol..

[66] Grobben G., Smith M., Sikkema J., De Bont J. (1996). Influence of fructose and glucose on the production of exopolysaccharides and the activities of enzymes involved in the sugar metabolism and the synthesis of sugar nucleotides in *Lactobacillus delbrueckii* subsp. *bulgaricus* NCFB 2772. Appl. Microbiol. Biotechnol..

[67] Franz C.M., Vancanneyt M., Vandemeulebroecke K., De Wachter M., Cleenwerck I., Hoste B., Schillinger U., Holzapfel W.H., Swings J. (2006). *Pediococcus stilesii* sp. nov., isolated from maize grains. Int. J. Syst. Evol. Microbiol..

[68] Orla-Jensen S. (1919). The Lactic Acid Bacteria.

[69] Dobson C.M., Deneer H., Lee S., Hemmingsen S., Glaze S., Ziola B. (2002). Phylogenetic analysis of the genus Pediococcus, including *Pediococcus claussenii* sp. nov., a novel lactic acid bacterium isolated from beer. Int. J. Syst. Evol. Microbiol..

[70] Russo P., de Chiara M.L.V., Capozzi V., Arena M.P., Amodio M.L., Rascón A., Dueñas M.T., López P., Spano G. (2016). *Lactobacillus plantarum* strains for multifunctional oat-based foods. LWT Food Sci. Technol..

[71] Rieder A., Ballance S., Knutsen S.H. (2015). Viscosity based quantification of endogenous β-glucanase activity in flour. Carbohydr. Polym..

[72] Vuyst D., de Ven V. (1998). Production by and isolation of exopolysaccharides from *Streptococcus thermophilus* grown in a milk medium and evidence for their growth-associated biosynthesis. J. Appl. Microbiol..

[73] Cerning J., Bouillanne C., Landon M., Desmazeaud M. (1992). Isolation and characterization of exopolysaccharides from slime-forming mesophilic lactic acid bacteria. J. Dairy Sci..

[74] Ricciardi A., Parente E., Crudele M.A., Zanetti F., Scolari G., Mannazzu I. (2002). Exopolysaccharide production by *Streptococcus thermophilus* SY: Production and preliminary characterization of the polymer. J. Appl. Microbiol..

[75] Banu I., Vasilean I., Aprodu I. (2011). Quality evaluation of the sourdough rye breads. Ann. Univ. Dunarea Jos Galati. Fascicle VI Food Technol..

[76] Lotong V., IV E.C., Chambers D.H. (2000). Determination of the sensory attributes of wheat sourdough bread. J. Sens. Stud..

[77] Messens W., De Vuyst L. (2002). Inhibitory substances produced by *Lactobacilli* isolated from sourdoughs—A review. Int. J. Food Microbiol..

[78] Korakli M., Pavlovic M., Ganzle M.G., Vogel R.F. (2003). Exopolysaccharide and kestose production by *Lactobacillus sanfranciscensis* LTH2590. Appl. Environ. Microbiol..

