# Supplementary material for: β-Glucan Production by Levilactobacillus brevis and Pediococcus claussenii for In Situ Enriched Rye and Wheat Sourdough Breads
Source: Foods. 2021 Mar 6;10(3):547. doi: 10.3390/foods10030547 (PMC7998486; doi:10.3390/foods10030547)
Supplement: Supplementary file 1 [file foods-10-00547-s001.zip › Table S2.docx]

Table S2. Results of rye sourdough analyses: cell count, pH values, MALDI-TOF MS and ratio of EPS positive colonies.

|  |  |  | ***L. brevis* TMW 1.2112** | | ***L. brevis* TMW 1.2320** | | ***P. claussenii* TMW 2.340** | | ***P. claussenii* TMW 2.2123** | |  |
| --- | --- | --- | --- | --- | --- | --- | --- | --- | --- | --- | --- |
|  |  |  | **Fermentation time [h]** | | | | | | | |  |
|  | **Temperature** | **Inoculum** | **0** | **24** | **0** | **24** | **0** | **24** | **0** | **24** |  |
| **Cell count**  **[CFU/g]** | 25 °C | 1 x | 5.6 · 10^5^ | 3.3 · 10^9^ | 3.4 · 10^5^ | 1.2 · 10^9^ | 2.9 · 10^6^ | 1.2 · 10^9^ | 2.6 · 10^6^ | 1.2 · 10^9^ |  |
|  | 28 °C | 1 x | 2.3 · 10^6^ | 3.4 · 10^9^ | 1.2 · 10^6^ | 3.0 · 10^9^ | 1.7 · 10^6^ | 1.4 · 10^9^ | 2.8 · 10^6^ | 1.9 · 10^9^ |  |
|  | 35 °C | 1 x | 1.6 · 10^6^ | 2.3 · 10^9^ | 1.3 · 10^6^ | 2.0 · 10^9^ | 3.3 · 10^6^ | 7.0 · 10^8^ | 3.0 · 10^6^ | 8.8 · 10^8^ |  |
|  | 28 °C | 1/2 x | 8.0 · 10^5^ | 2.6 · 10^9^ | 7.2 · 10^5^ | 2.4 · 10^9^ | 1.6 · 10^6^ | 1.4 · 10^9^ | 1.1 · 10^6^ | 1.4 · 10^9^ |  |
|  | 28 °C | 2 x | 3.3 · 10^6^ | 4.0 · 10^9^ | 2.7 · 10^6^ | 2.1 · 10^9^ | 6.6 · 10^6^ | 7.9 · 10^8^ | 4.3 · 10^6^ | 1.0 · 10^9^ |  |
|  | 28 °C | 1 x (Coculture) | 1.3 · 10^5^ | 2.4 · 10^9^ | 1.7 · 10^5^ | 1.9 · 10^9^ | 2.9 · 10^5^ | 2.2 · 10^9^ | 3.1 · 10^5^ | 1.7 · 10^9^ |  |
| **pH [-]** | 25 °C | 1 x | 5.78 ± 0.01 | 3.79 ± 0.01 | 5.80 ± 0.01 | 3.78 ± 0.01 | 5.81 ± 0.03 | 3.92 ± 0.01 | 5.81 ± 0.01 | 3.86 ± 0.01 |  |
|  | 28 °C | 1 x | 5.86 ± 0.01 | 3.68 ± 0.01 | 5.83 ±0.02 | 3.67 ± 0.01 | 5.77 ± 0.05 | 3.77 ± 0.02 | 5.79 ± 0.03 | 3.76 ± 0.02 |  |
|  | 35 °C | 1 x | 5.78 ± 0.01 | 3.58 ± 0.01 | 5.80 ± 0.01 | 3.58 ± 0.01 | 5.81 ± 0.01 | 3.75 ± 0.01 | 5.80 ± 0.02 | 3.70 ± 0.01 |  |
|  | 28 °C | 1/2 x | 5.76 ± 0.01 | 3.70 ± 0.01 | 5.75 ± 0.01 | 3.71 ± 0.01 | 5.75 ± 0.01 | 3.81 ± 0.00 | 5.74 ± 0.01 | 3.80 ± 0.01 |  |
|  | 28 °C | 2 x | 5.73 ± 0.02 | 3.69 ± 0.01 | 5.74 ± 0.01 | 3.68 ± 0.01 | 5.72 ± 0.01 | 3.75 ± 0.01 | 5.86 ± 0.01 | 3.70 ± 0.00 |  |
|  | 28 °C | 1 x (Coculture) | 5.74 ± 0.01 | 3.61 ± 0.01 | 5.75 ± 0.00 | 3.61 ± 0.01 | 5.74 ± 0.01 | 3.60 ± 0.01 | 5.72 ± 0.02 | 3.61 ± 0.01 |  |
| **Inoculated species identity [%]** | 25 °C | 1 x | 100 | 100 | 100 | 100 | 98 | 99 | 99 | 100 |  |
|  | 28 °C | 1 x | 96 | 100 | 96 | 100 | 96 | 100 | 92 | 100 |  |
|  | 35 °C | 1 x | 100 | 100 | 98 | 93 | 99 | 99 | 96 | 100 |  |
|  | 28 °C | 1/2 x | 99 | 100 | 98 | 100 | 96 | 100 | 97 | 100 |  |
|  | 28 °C | 2 x | 100 | 100 | 100 | 100 | 99 | 100 | 98 | 97 |  |
|  | 28 °C | 1 x (Coculture) | 48 | 48 | 47 | 40 | 66 | 70 | 65 | 21 |  |
| **EPS positive CFU [%]** | 25 °C | 1 x | 100 | 100 | 0 | 0 | 97 | 98 | 0 | 0 |  |
|  | 28 °C | 1 x | 100 | 100 | 0 | 0 | 16 | 38 | 0 | 0 |  |
|  | 35 °C | 1 x | 100 | 100 | 0 | 0 | 98 | 95 | 0 | 0 |  |
|  | 28 °C | 1/2 x | 100 | 100 | 0 | 0 | 50 | 34 | 0 | 0 |  |
|  | 28 °C | 2 x | 100 | 100 | 0 | 0 | 48 | 42 | 0 | 0 |  |
|  | 28 °C | 1 x (Coculture) | 100 | 100 | 0 | 0 | 27 | 5 | 0 | 0 |  |
