# Supplementary material for: β-Glucan Production by Levilactobacillus brevis and Pediococcus claussenii for In Situ Enriched Rye and Wheat Sourdough Breads
Source: Foods. 2021 Mar 6;10(3):547. doi: 10.3390/foods10030547 (PMC7998486; doi:10.3390/foods10030547)
Supplement: Supplementary file 1 [file foods-10-00547-s001.zip › Table S1.docx]

Table S1. Results of wheat sourdough analyses: cell count, pH values, MALDI-TOF MS and ratio of EPS positive colonies.

|  |  |  | ***L. brevis* TMW 1.2112** | | ***L. brevis* TMW 1.2320** | | ***P. claussenii* TMW 2.340** | | ***P. claussenii* TMW 2.2123** | |  |
| --- | --- | --- | --- | --- | --- | --- | --- | --- | --- | --- | --- |
|  |  |  | **Fermentation time [h]** | | | | | | | |  |
|  | **Temperature** | **Inoculum** | **0** | **24** | **0** | **24** | **0** | **24** | **0** | **24** |  |
| **Cell count**  **[CFU/g]** | 25 °C | 1 x | 6.6 · 10^5^ | 9.0 · 10^8^ | 7.5 · 10^5^ | 4.1 · 10^8^ | 2.8 · 10^6^ | 8.4 · 10^8^ | 3.0 · 10^6^ | 6.6 · 10^8^ |  |
|  | 28 °C | 1 x | 2.3 · 10^6^ | 1.0 · 10^9^ | 2.0 · 10^6^ | 1.0 · 10^9^ | 4.0 · 10^6^ | 6.4 · 10^8^ | 3.3 · 10^6^ | 6.9 · 10^8^ |  |
|  | 35 °C | 1 x | 1.5 · 10^6^ | 1.2 · 10^9^ | 1.5 · 10^6^ | 1.3 · 10^9^ | 3.2 · 10^6^ | 3.3 · 10^8^ | 3.7 · 10^6^ | 4.2 · 10^8^ |  |
|  | 28 °C | 1/2 x | 8.6 · 10^5^ | 6.5 · 10^8^ | 6.8 · 10^5^ | 8.4 · 10^8^ | 1.3 · 10^6^ | 6.7 · 10^8^ | 1.2 · 10^6^ | 7.4 · 10^8^ |  |
|  | 28 °C | 2 x | 3.0 · 10^6^ | 6.6 · 10^8^ | 3.6 · 10^6^ | 7.2 · 10^8^ | 6.5 · 10^6^ | 8.1 · 10^8^ | 5.7 · 10^6^ | 6.6 · 10^8^ |  |
|  | 28 °C | 1 x (Coculture) | 1.5 · 10^5^ | 1.7 · 10^9^ | 1.6 · 10^5^ | 1.6 · 10^9^ | 2.3 · 10^5^ | 1.1 · 10^9^ | 2.7 · 10^5^ | 1.6 · 10^9^ |  |
| **pH [-]** | 25 °C | 1 x | 5.98 ± 0.01 | 3.94 ± 0.02 | 5.99 ± 0.01 | 3.92 ± 0.02 | 6.02 ± 0.01 | 3.76 ± 0.01 | 6.04 ± 0.01 | 3.74 ± 0.01 |  |
|  | 28 °C | 1 x | 6.02 ± 0.01 | 3.69 ± 0.01 | 6.01 ± 0.01 | 3.69 ± 0.01 | 5.95 ± 0.04 | 3.67 ± 0.01 | 5.97 ± 0.05 | 3.68 ± 0.01 |  |
|  | 35 °C | 1 x | 5.97 ± 0.02 | 3.50 ± 0.01 | 5.96 ± 0.01 | 3.51 ± 0.01 | 5.99 ± 0.02 | 3.62 ± 0.02 | 5.99 ± 0.01 | 3.59 ± 0.01 |  |
|  | 28 °C | 1/2 x | 5.98 ± 0.00 | 3.72 ± 0.01 | 5.97 ± 0.01 | 3.71 ± 0.01 | 5.97 ± 0.01 | 3.68 ± 0.01 | 5.97 ± 0.01 | 3.68 ± 0.01 |  |
|  | 28 °C | 2 x | 5.96 ± 0.01 | 3.70 ± 0.02 | 5.97 ± 0.02 | 3.70 ± 0.01 | 5.95 ± 0.01 | 3.65 ± 0.00 | 5.96 ± 0.01 | 3.64 ± 0.01 |  |
|  | 28 °C | 1 x (Coculture) | 5.99 ± 0.01 | 3.57 ± 0.01 | 5.96 ± 0.01 | 3.56 ± 0.01 | 5.95 ± 0.01 | 3.56 ± 0.01 | 5.95 ± 0.01 | 3.55 ± 0.01 |  |
| **Inoculated species identity [%]** | 25 °C | 1 x | 93 | 100 | 94 | 100 | 100 | 98 | 100 | 100 |  |
|  | 28 °C | 1 x | 100 | 100 | 91 | 100 | 94 | 100 | 72 | 100 |  |
|  | 35 °C | 1 x | 99 | 99 | 92 | 100 | 100 | 100 | 100 | 100 |  |
|  | 28 °C | 1/2 x | 100 | 100 | 100 | 100 | 100 | 100 | 99 | 100 |  |
|  | 28 °C | 2 x | 100 | 100 | 100 | 100 | 100 | 100 | 99 | 100 |  |
|  | 28 °C | 1 x (Coculture) | 45 | 26 | 38 | 21 | 50 | 61 | 60 | 21 |  |
| **EPS positive CFU [%]** | 25 °C | 1 x | 100 | 100 | 0 | 0 | 99 | 96 | 0 | 0 |  |
|  | 28 °C | 1 x | 100 | 100 | 0 | 0 | 39 | 42 | 0 | 0 |  |
|  | 35 °C | 1 x | 100 | 100 | 0 | 0 | 97 | 95 | 0 | 0 |  |
|  | 28 °C | 1/2 x | 100 | 100 | 0 | 0 | 48 | 41 | 0 | 0 |  |
|  | 28 °C | 2 x | 100 | 100 | 0 | 0 | 57 | 36 | 0 | 0 |  |
|  | 28 °C | 1 x (Coculture) | 100 | 100 | 0 | 0 | 32 | 8 | 0 | 0 |  |
